# A programmable nanoreplica molding for the fabrication of nanophotonic devices

**DOI:** 10.1038/srep22445

**Published:** 2016-03-01

**Authors:** Longju Liu, Jingxiang Zhang, Mohsin Ali Badshah, Liang Dong, Jingjing Li, Seok-min Kim, Meng Lu

**Affiliations:** 1 Department of Electrical and Computer Engineering, Iowa State University, Ames, Iowa 50011, USA; 2 School of Mechanical Engineering, Chung-Ang University, Seoul 156-756, Republic of Korea; 3 Department of Mechanical Engineering, University of Hawaii, Honolulu, Hawaii 96822, USA; 4 Department of Mechanical Engineering, Iowa State University, Ames, Iowa 50011, USA

## Abstract

The ability to fabricate periodic structures with sub-wavelength features has a great potential for impact on integrated optics, optical sensors, and photovoltaic devices. Here, we report a programmable nanoreplica molding process to fabricate a variety of sub-micrometer periodic patterns using a single mold. The process utilizes a stretchable mold to produce the desired periodic structure in a photopolymer on glass or plastic substrates. During the replica molding process, a uniaxial force is applied to the mold and results in changes of the periodic structure, which resides on the surface of the mold. Direction and magnitude of the force determine the array geometry, including the lattice constant and arrangement. By stretching the mold, 2D arrays with square, rectangular, and triangular lattice structures can be fabricated. As one example, we present a plasmonic crystal device with surface plasmon resonances determined by the force applied during molding. In addition, photonic crystal slabs with different array patterns are fabricated and characterized. This unique process offers the capability of generating various periodic nanostructures rapidly and inexpensively.

Periodic nanostructures, such as 1D and 2D sub-wavelength gratings, are critical to a broad range of optical applications as they control light propagation and can enhance light-matter interactions^1^. They are exploited in various photonic devices, including diffraction gratings, wire grid polarizers, grating couplers, distributed feedback lasers, and photonic crystals^2^,^3^,^4^,^5^. Fabrication of periodic nanostructures has been limited by the need to work on a sub-micrometer scale; conventional lithography methods, such as using e-beam and deep ultraviolet, are either too expensive or have insufficient throughput for wafer-scale fabrications. To address this issue, interference lithography and soft lithography have been successfully applied^6^,^7^,^8^,^9^,^10^,^11^. Soft lithography provides the capability of inexpensive, roll-to-roll fabrication of periodic nanostructures for example, the pattern of a mold can be transferred to a photo-curable polymer material at room temperature by the nanoreplica molding process, without the need for large mechanical forces^12^,^13^,^14^.

Although soft lithography has been successful, a drawback is the high cost of molds. To introduce or modify a feature, a new mold must be fabricated. To facilitate the production of different patterns, and periodic structures in particular, programmable approaches have been developed. For example, thermal tuning of a thermoplastic substrate bearing a nanopattern can generate a variety of patterns from a single mold^15^. Alternatively, Pokroy *et al.* took advantage of elastomeric and flexible molds to generate arrays of nanoposts with a variety of micrometer-scale periods^16^.

In this paper, we present various plasmonic crystals and photonic crystal slabs fabricated by a programmable nanoreplica molding process that uses a single mold. This technique uses mechanical stretching of an elastic polydimethylsiloxane (PDMS) mold to create periodic structures with various periods and lattice arrangements. At the same time, it maintains the high-throughput and low-cost features of the conventional nanoreplica molding approach. When a force is applied, the surface of the PDMS mold adjusts itself to a negative volume profile of the desired periodic structure. Replicating the stretched mold shape in a UV-curable polymer (UVCP) yields programmable nanostructures in a process that is inexpensive and amenable to scale-up. Following replica molding, the periodic structures produced may be coated with a dielectric or thin metal film; some examples are titanium dioxide (TiO_2_), gold, and silver. With a 100 nm-thick silver coating, the fabricated 2D plasmonic crystals exhibit surface plasmon resonances (SPRs) in the spectral range 410 nm to 570 nm. Using the same PDMS mold, we fabricated photonics crystal slabs with three different lattice arrangements, namely, square, rectangular, and triangular structures. The photonic crystal slabs use a 160 nm-thick TiO_2_ film as the light-confinement layer. Finally, band diagrams of the fabricated photonic crystal slabs are experimentally determined and compared to simulations from electromagnetic theory.

## Results and Discussion

**Figure 1:**
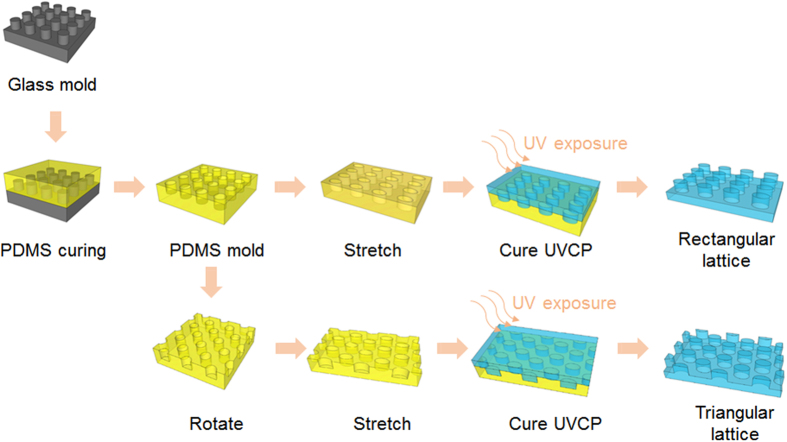
Schematic of the programmable nanoreplica molding process.

Figure 1 summarizes the major steps of the programmable nanoreplica molding process. The major fabrication procedures include mold preparation, mold stretching, pattern transfer, and mold release. As the initial step, a PDMS mold is replicated from a rigid glass stamp carrying a 2D array of nanoposts with a square lattice arrangement and periods *Λ*_*x*_ = *Λ*_*y*_ = 300 nm. The glass stamp was produced using a glass thermal imprinting process, reported previously^17^,^18^. Next, the PDMS mold is precisely stretched to obtain a lattice pattern for a particular device. A uniaxial force is applied to the PDMS mold in the plane parallel to its surface. Along the direction of the force (*y*-axis), the array is stretched and its period increases. Consequently, the array is also compressed in the perpendicular *x*-direction and the array period is reduced. Likewise, stretching the PDMS mold in other directions also allows us to program the lattice arrangement. As shown in Fig. 1, the PDMS mold can be pulled along a diagonal direction to convert the original square lattice into a triangular lattice. From a stretched PDMS mold, the modified periodic pattern is replicated onto a glass or plastic substrate by the nanoreplica molding process. The details of the process are described below in the experimental section. Briefly, a layer of liquid UVCP material is squeezed between the stretched PDMS mold and a glass or plastic substrate. On exposure to UV illumination, the UVCP solidifies and subsequently releases from the PDMS mold.

**Figure 2:**
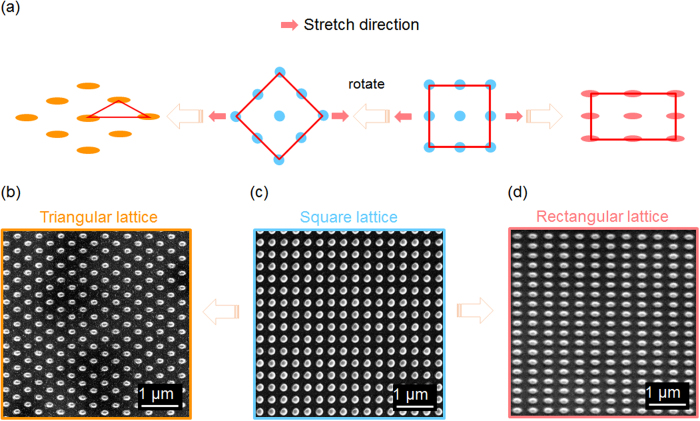
Nanopost arrays replicated using a single PDMS mold. Scheme of the mold programming process, beginning at the third figure from the left (**a**). SEM images of nanopost arrays with triangular lattice (**b**), square lattice (**c**), and rectangular lattice (**d**).

Figure 2(a) schematically illustrates how the geometry of the periodic nanostructure is tuned to produce different types of arrays. The blue dots represent the nanoposts of the un-stretched square lattice with a period of 300 nm. When a uniaxial force is applied along the *x*-axis, the square lattice is changed to a rectangular one, as shown by the array of cherry-colored dots. Figure 2(b–d) are the scanning electron microscopy (SEM) images of replicated triangular, square, and rectangular arrays, respectively. The rectangular array shown in Fig. 2(d) was fabricated when the PDMS mold was under a strain of 40%. The period of the array increases to *Λ*_*x*_ = 420 nm along the direction of the applied force. The lattice shows a slight shrinkage in the direction perpendicular to the force, with a period of *Λ*_*y*_ = 258 nm. Meanwhile, the cross section of the nanoposts changes from circular to elliptical; however the duty cycles of the periodic structure (post size/period) remain unchanged. When the PDMS mold is stretched along the diagonal direction, it is possible to produce periodic arrays with a triangular lattice, which is illustrated by the array of orange dots in Fig. 2(b). Figure 2(b) shows the replication fabricated when the PDMS mold was under a 40% stretch along the 45° direction to the *x*-axis. With precise control of the degree and direction of the stretch, the triangular lattice could be tuned into a hexagonal one.

**Figure 3:**
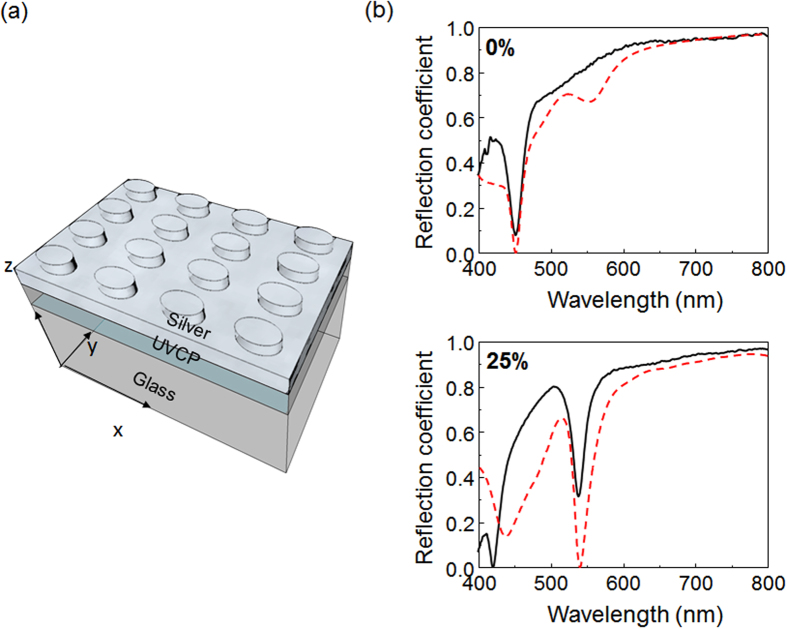
Optical characterization of 2D plasmonic crystals. (**a**) Schematic of a silver-coated plasmonic slab. (**b**) Simulations for 0% and 25% strain, compared to experimental data. The black solid lines represent the measurement results, and the red dashed lines represent the simulations.

The capability of programming the array lattice is particularly useful for some nanophotonic devices. As an example, we studied plasmonic crystals with different periodic arrays fabricated using the process described above. As a result of the different geometries, these arrays exhibit distinct plasmonic resonances. The plasmonic crystal structure, shown in Fig. 3(a), consists of a replicated 2D array of nanoposts with a 100 nm-thick silver coating, and supports grating- coupled surface plasmon resonance (SPR) modes. Excitation light that meets the resonance condition can be coupled into an SPR mode and may be strongly absorbed. As a result, the reflection spectrum exhibits a dip with the minimum reflectance at the SPR wavelength (*λ*_*SP*_). The resonance wavelength can be estimated using the equation:


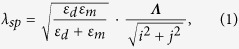


where *Λ* is the period of the grating, (*i*, *j*) represent the Bragg resonance orders, and *ε*_*m*_ and *ε*_*d*_ are the dielectric constants of metal and surrounding medium, respectively^19^,^20^. Because the SPRs of plasmonic crystals are determined by the grating period shown in Eq. 1, the programmable replica molding process can be used to finely tune the periodic array and obtain the desired SPR mode wavelength.

**Figure 4:**
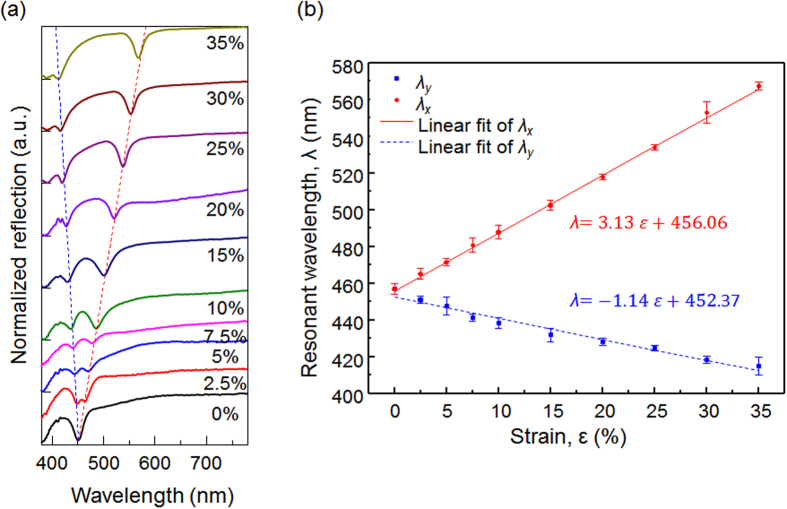
(**a**) The SPR reflection dips for plasmonic slabs fabricated with 0%, 2.5%, 5%, 7.5%, 10%, 15%, 20%, 25%, 30%, and 35% strain. The split between the dips for x- and y-polarizations follows the lattice period deviations in the *x*-direction (elongation) and *y*-direction (compression). (**b**) Plasmonic resonance wavelength with respect to applied strain. The SPR dips in the stretched and compressed directions from the experimental data in (**a**) are shown by the red dots and blue squares, respectively; linear fits to the resonance wavelengths, by the solid red and dashed blue lines. The error bars represent the standard deviation of 10 measurements taken at different locations on the sample.

In fact, the resonance wavelength varies proportionally to the strain (*∈*) that is generated in the PDMS mold by the uniaxial stretch. In order to experimentally investigate the correlation between *λ*_*SP*_ and *ϵ*, we replicated 10 different arrays of nanoposts by placing the PDMS mold under a range of strains (*∈* = 0%, 2.5%, 5%, 7.5%, 10%, 15%, 20%, 25%, 30%, and 35%). The replicated 2D gratings were subsequently coated with a 100 nm-thick silver film to form the plasmonic crystals. The same PDMS mold was reused 10 times to produce the plasmonic devices. Reflection spectra of the plasmonic crystals were measured to identify their resonance wavelengths. Rigorous coupled wave analysis (RCWA) was used to simulate these reflection spectra. Details of the simulation and measurement are discussed in the experimental section. Figure 3(b) compares the measured and simulated reflection spectra of devices fabricated with 0% and 25% strain. With 0% strain, the device exhibits a reflection dip at *λ*_*SP*_ = 456 nm; the *x*- and *y*-polarized SPR modes coincide in wavelength because the array structure is symmetric. As shown in Fig. 4(a), the stretch applied during molding splits the differently polarized resonance modes. The SPR mode with the electric field polarized along the *x*-axis shifts to longer wavelengths with increased stretch; the shift is proportional to the degree of stretching. The corresponding compression along the *y*-axis causes the *y*-polarized SPR mode to move to shorter wavelengths. For *ϵ* = 35%, the plasmonic resonances are shifted by 111 nm and −41 nm for the *x*- and *y*- polarizations, respectively. Figure 4(b) summarizes *λ*_*SP*_ as a function of strain for both polarizations. The measured resonance wavelengths are fitted by straight lines with slopes of *λ*_*SP*_/*ϵ* = 3.13 and −1.14 nm/(% strain) for the *x*- and *y*-polarizations, respectively.

**Figure 5:**
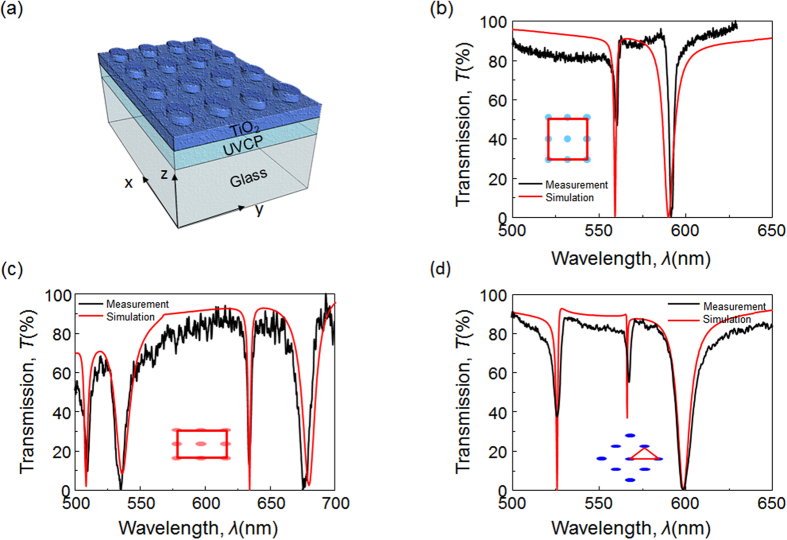
Reflection spectra of plasmonic crystals. (**a**) Schematic diagram of a photonic crystal slab with a rectangular lattice. Experimental and simulated transmission spectra of photonic crystal slabs when the resonance modes are excited from the normal direction, for a square (**b**), rectangular (**c**), and triangular lattice (**d**).

The array control in the nanoreplica molding method can also be used to examine and modify the photonic band diagram of photonic crystal slabs. In this study, the photonic crystal slab is based on a grating coupled waveguide, which is also known as a leaky-mode waveguide. The guided-mode resonance (GMR) phenomenon supported by this structure provides narrowband optical resonances^3^,^21^,^22^. As shown in Fig. 5(a), the photonic crystal slab consists of a replicated 2D grating on a glass substrate, which can couple light into and out of a thin-film dielectric waveguide coated on the surface of the grating. At a specific combination of wavelength and angle of incidence, the GMR mode can be excited with nearly 100% reflectance. Based on the diffraction grating equation, the GMR wavelength and the resonant angle can be calculated from the equations: Finally, *n*_1_, *n*_2_, and *n*_3_ represent the refractive indices of the superstrate, the grating layer material, and the substrate, respectively^23^,^24^. Like plasmonic crystals, the resonance characteristics of a photonic crystal slab also depend on the array geometry.

Here, we demonstrate photonic crystal slabs with three different lattice arrangements, namely, the square, the rectangular, and the triangular lattice. The photonic crystal slabs were fabricated using the programmable replica molding approach, followed by coating with a 160 nm-thick TiO_2_ layer (refractive index *n* = 2.0). The transmission spectra were measured using broadband light from the direction normal to the sample surface. As shown in Fig. 5(b), the photonic crystal slab with a square lattice (*Λ*_*x*_ = *Λ*_*y*_ = 300 nm) shows two GMR modes at 560.2 nm and 591.6 nm, representing the TM-polarized and TE-polarized modes, respectively. For the TM-polarized modes, the electric field components are perpendicular to the periodic modulation, while for the TE-polarized modes, they are parallel to the modulation. Because of the symmetry of the square lattice, the reflection measurement is independent of the excitation light polarization. Figure 5(b) also compares the measurement to the RCWA simulation. The discrepancy between measured and simulated resonance characteristics likely arises because the simulation does not take into account the slight divergence of the incident light.

To generate a rectangular lattice, the PDMS mold was stretched with a uniaxial force along the *x*-axis to generate a strain of 25%. The replicated array structure shows two distinct periods (*Λ*_*x*_ = 375 nm and *Λ*_*y*_ = 280 nm) along the *x-*axis and *y-*axis, respectively. As shown in Fig. 5(c), the rectangular lattice slab exhibits two TM-polarized modes at 634.5 nm and 510.1 nm corresponding to the modified array periods. At the same time, there are also two TE-polarized modes locating at 677.2 nm and 535.5 nm. Next, to fabricate a photonic crystal slab with a triangular lattice (Fig. 2(d)), the PDMS mold was stretched along its diagonal direction to achieve a strain of 35%. The measured transmission of the triangular lattice photonic crystal is shown in Fig. 5(d). For comparison, the red curves in Fig. 5(c,d) represent the transmission spectra calculated using an RCWA simulation.

**Figure 6:**
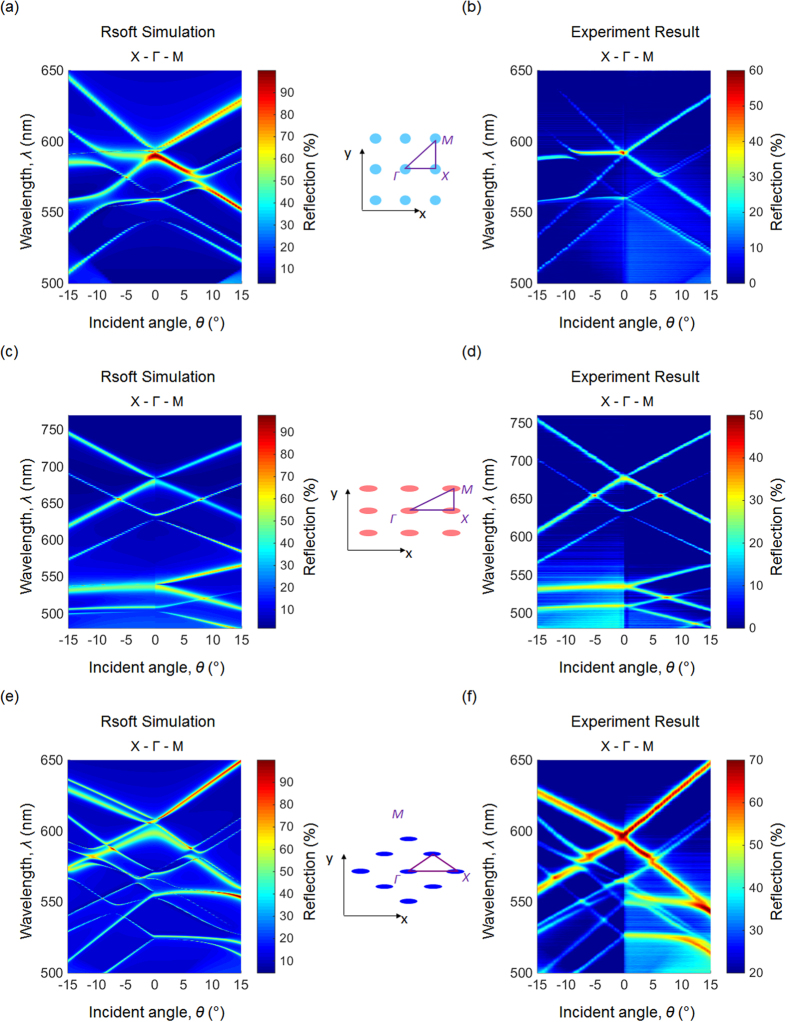
Photonic band diagrams derived from the transmission spectra. (**a,b**) Simulated and measured photonic band diagrams for the square lattice structure. (**c,d**) Simulated and measured photonic band diagrams for the rectangular lattice structure. (**e,f**) Simulated and measured photonic band diagrams for the triangular lattice structure.

To further investigate the effect of lattice arrangement, the dispersion of GMR modes as a function of incident angle was studied. Transmission spectra were recorded when the incident angle was scanned along the high-symmetry directions, as described in the experimental section. For the square lattice PC slab, the measured and simulated photonic band structures are shown in Fig. 6(a). When the angle of incidence (*θ*_*x*_) increases from the Γ point toward the X point, both TE-polarized and TM-polarized modes form three bands. The center “flat” band represents the GMR coupled with the excitation light through the grating modulation along the *y*-direction. In contrast, the GMR modes coupled through the grating modulation along the *x*-direction split into two bands, an upper band and a lower band with respect to their resonant wavelength. The lower TE-polarized band and upper TM-polarized band intersect at *θ*_*x*_ = 15°. When the angle of incidence changes along the Γ-M, the TE-polarized GMR modes form an upper band and a lower band. The TM-polarized modes branch into two upper bands and two lower bands.

Figure 6(b) shows the measured and simulated band structures for the rectangular photonic crystal slab with the angle of incidence scanned from the Γ point to the X and M points, respectively. When the angle of incidence increases from the Γ point towards the X point, two flat bands (one for a TE band and one for a TM) are observed. The flat bands are the GMR modes coupled *via* the grating modulation along the *y*-direction. The GMR modes coupled *via* the *x*-direction modulation are located in a longer wavelength region because the rectangular grating is not symmetric and *Λ*_*x*_ is larger than *Λ*_*y*_. For both TE and TM polarizations, the GMR modes split into an upper band and a lower band. From the Γ point to the M point, all four GMR modes form two bands, resulting in eight different bands. Likewise, Fig. 6(c) shows the experimental and calculated photonic band diagrams for the device with a triangular array, where the angle of incidence is scanned from the Γ point to the X and M points.

## Conclusion

In summary, this paper reports a programmable nanoreplica molding method that facilitates fabrication of grating-based nanophotonic devices. Using a single PDMS mold, this fabrication method can produce sub-wavelength structures with various lattices. We adopted the stretchable PDMS mold, which was replicated from a glass stamp, to fabricate periodic nanostructures using UVCP material on a glass or a plastic substrate. During nanoreplica molding, the PDMS mold was stretched precisely to produce the desired lattice geometry. Nanophotonic devices, including plasmonic and photonic crystals, were formed by coating metal or dielectric thin films on the replicated UVCP. The optical resonances of these devices were characterized experimentally and compared to simulation results. For the plasmonic crystals, an SPR wavelength of 456 nm increased to 566 nm with application of 35% uniaxial strain during molding. The range of the resonance wavelengths could be even greater with increased stretch, since the PDMS can endure as high as 100% strain^25^,^26^. For the photonic crystal slabs, reflection filters with three different lattice geometries were fabricated. Rectangular and triangular lattices were successfully obtained from the original square lattice. The photonic band diagrams of all three devices were measured and showed good agreement with simulations.

## Materials and Methods

### Nanoreplica molding process

The master stamp with a 2D square array (*Λ* = 300 nm) of 150 nm diameter posts and an overall dimension of 50 mm × 50 mm was fabricated using the glass thermal imprinting method with vitreous mold prepared by carbonization of replicated furan precursor^17^,^18^. The glass master stamp was cleaned and treated using an anti-adhesion silane (Repel Silane, GE Healthercare) in order to facilitate the replications. The PDMS mold was fabricated from the master stamp by the thermally curing of a mixture of PDMS elastomer and curing agent (in a volume ratio of 1:10) on the master stamp. The thickness of PDMS molds was controlled to be 2 mm. After curing at 100 °C for 4 hours, the solidified PDMS was peeled away from the master stamp. Then the PDMS mold was cut into a rectangle that has a length of 50 mm and a width of 15 mm. The orientation of the lattice on the PDMS mold was identified and marked. During the UV-based replica molding process, the PDMS mold was placed between two grips that were separated by *L* ~ 45 mm on a customized stage. One of the grips was fixed and the other one was pulled horizontally (along *x*-direction) by a linear translation stage. The translation stage induced strain values, ε_x_ = Δ*x*/*L*, where Δ*x* is the amount of stretch. The PDMS mold was held at the desired length during the replica molding process. A layer of liquid UVCP (NOA 88, Norland Product Inc.) was squeezed between the PDMS mold and a glass coverslip. The UV-curing process took place by exposing the coverslip/liquid UVCP/PDMS stack to UV illumination for 300 s. After curing, the replica of a 2D array of nanoposts and the PDMS mold were separated by peeling the coverslip away from the PDMS mold. The fully cured polymer preferentially adheres to the glass substrate without leaving any residue on the PDMS mold. Subsequent to the replica molding, a layer of dielectric (TiO_2_) or silver thin film was deposited over the surface relief 2D grating by electron-beam evaporation to complete the device fabrication.

### Reflection measurement for plasmonic crystals

Reflection spectra of plasmonic crystals were measured using a white light reflection setup. A halogen lamp was used as a broadband excitation source, and was coupled into a bifurcated fiber (BFY50HS02, Thorlabs), with a fiber tip collimator at the exit. An iris and a linear polarizer were placed in front of the collimator to control the spot size and polarization of the incident beam. The illumination assembly was attached to a kinematic mount for precise adjustment of the angle of incidence. The reflected light was coupled into a spectrometer (USB2000, OceanOptics) through the same bifurcated fiber. For measurement of its reflection spectrum, a plasmonic crystal sample was mounted on a motorized *x-y* translation stage and immersed in deionized water. A silver-coated mirror was used as the reference for reflectance. Software developed using C# was used to control movement of the translation stage and collect spectra from the spectrometer for chosen sampling locations on the plasmonic crystals. The measured spectra were fitted using a second-order polynomial function to find the resonance wavelength of the plasmonic modes.

### Photonic crystal transmission and band diagram measurements

The dispersion band diagrams of the photonic crystal slabs were measured by recording the transmission spectra for multiple incident angles. The experimental setup for transmission measurement has three main parts: a halogen lamp for white broadband illumination, a sample mount, and a spectrometer. The white light was collimated before exiting the coupling fiber. The light beam passed through the photonic crystal slab and the transmitted light was collected using a multimode fiber collector, which was connected to the spectrometer. The instruments were aligned horizontally and the plane of incidence was chosen to be the horizontal plane. The sample mount was carefully designed, with two perpendicular rotation stages and a kinematic mount, providing sufficient degrees of freedom for complicated and precise angle adjustments. At the beginning of each measurement, a sample was mounted with its top face perpendicular to the incoming light (at the Γ-Point). Then, based on the direction to be measured, i.e., Γ-X or Γ-M, the sample was rotated vertically so that the direction being measured was parallel with the horizontal plane. Next, the sample was rotated horizontally so that the incident angle *θ* was scanned accordingly from 0° to 15° by increments of 0.5°. For each *θ* the transmission spectrum was recorded, for both Γ-X and Γ-M directions, to form a data cube. The photonic band diagram was plotted using *θ* and *λ* as *x*-axis and *y*-axis, respectively.

### Electromagnetic modeling

The RCWA simulations were performed to model the reflection and transmission spectra of the plasmonic crystals and photonic crystal slabs. The simulation region was setup to the unit volume of the periodic structures. Periodic boundary conditions were applied to truncate the calculation domain in *x-y* plane. Ten spatial harmonics were used at both *x*- and *y*-directions. For plasmonic crystals, the devices were illuminated from the direction normal to the surface by a plane wave. The incident wave was linearly polarized with the electric field oriented at 45° to *x*-axis in *x-y* plane. The material properties of silver were taken from the Palik’s handbook, and then fitted by the multi-coefficient model in the wavelength range from 300 nm to 1000 nm. Reflection spectra of plasmonic crystals were calculated in the wavelength range from 375 nm to 775 nm. The simulation of photonic crystal slabs generated their dispersion diagrams. The transmission spectra of a photonic crystal slab were recorded when the angle of incidence (*θ*) was scanned from 0° to 15° with an incremental of 0.5°. The dispersion diagrams for the Γ-X directions were shown by plotting the transmission spectra as a function of *θ* when φ = 0°. The dispersion diagrams for the Γ-M directions were plotted when *φ* = 45°, 37.12°, and 90°, for the square, the rectangular, and the triangular lattices, respectively.

## Additional Information

**How to cite this article**: Liu, L. *et al.* A programmable nanoreplica molding for the fabrication of nanophotonic devices. *Sci. Rep.* **6**, 22445; doi: 10.1038/srep22445 (2016).
